# Investigating the Effect of Alcohol Dehydrogenase Gene Knockout on Lipid Accumulation in *Mucor circinelloides* WJ11

**DOI:** 10.3390/jof8090917

**Published:** 2022-08-29

**Authors:** Aabid Manzoor Shah, Hassan Mohamed, Abu Bakr Ahmad Fazili, Wu Yang, Yuanda Song

**Affiliations:** 1 Colin Ratledge Center of Microbial Lipids, School of Agriculture Engineering and Food Science, Shandong University of Technology, Zibo 255000, China; 2 Department of Botany and Microbiology, Faculty of Science, Al-Azhar University, Assiut 71524, Egypt

**Keywords:** *Mucor circinelloides* WJ11, alcohol dehydrogenase, Knockout *adh1∆*, ethanol, lipid biosynthesis

## Abstract

*Mucor circinelloides* is an oleaginous, dimorphic zygomycete fungus species that produces appreciable levels of ethanol when grown under aerobic conditions in the presence of high glucose, indicating the fungus is a Crabtree-positive microorganism. Engineering efforts to redirect carbon flux from ethanol to lipid biosynthesis may shed light on the critical role of ethanol biosynthesis during aerobic fermentation in *M. circinelloides*. Therefore, in this study, the alcohol dehydrogenase gene (*ADH1*) of *M. circinelloides* WJ11 was deleted, and its effects on growth, lipid production, and fatty acid content were analyzed. Our results showed that knocking out of *adh1∆* reduced the ethanol concentration by 85–90% in fermented broth, indicating that this gene is the major source of ethanol production. Parallel to these findings, the lipid and fatty acid content of the mutant was decreased, while less change in the growth of WJ11 was observed. Furthermore, a fermentation study showed the lipid and fatty acid content was restored in the mutant strain when the fermentation media was supplemented with 0.5% external ethanol, indicating the importance of alcohol dehydrogenase and its product on growth and lipid biosynthesis in *M. circinelloides*. To our knowledge, this is the first study to show a link between alcohol dehydrogenase and lipid production in *M. circinelloides*.

## 1. Introduction

*Mucor circinelloides* is an important organism in the history of microbial lipids that have been successfully used for the production of γ-linolenic acid (GLA) [1,2]. GLA—a member of the n-6 fatty acid family—serves as a substrate for the synthesis of eicosanoids, transporting, and oxidation of cholesterol, and forming components of the lipid membrane [3]. Many inflammatory and degenerative diseases in the human body are caused by insufficient dietary intake or impaired formation of GLA [3]. The goal of commercial microbial GLA production was to create a cheaper alternative to the GLA extracted from the seeds of evening primrose (*Oenothera biennis*), which was the only source of GLA [4]. The prospects for reviving the Mucor-derived GLA are extremely important at this point. *M. circinelloides*, an oleaginous fungus, was widely used to investigate GLA production and was chosen as a model to study microbial lipids [5,6]. *M. circinelloides* WJ11 was recently isolated and identified as a high lipid producer, capable of accumulating up to 36% lipid of its dry cell weight [7]. *M. circinelloides*’ lipid biosynthetic pathway is controlled by simple metabolic regulation, allowing for more modulation via fermentation conditions and genetic engineering. The availability of genomic data and easy-to-use genetic tools has made it easier to manipulate this strain, making it an important organism for microbial lipid research [8].

*M. circinelloides,* a dimorphic Zygomycete fungus, produces a significant amount of ethanol when grown under aerobic conditions in the presence of high glucose, indicating that it is a Crabtree-positive microorganism [9]. The ethanol produced by the fungus under these conditions is consumed after the glucose has been exhausted. Yeast cells grown in anaerobic conditions, on the other hand, do not use ethanol because they require hexoses as a carbon source [10]. Alcohol dehydrogenases (ADH) are widespread in the various microbes, their type and number vary in different species [11,12]. The kinetic analysis of purified ADH1 indicates that it primarily functions as a fermentative enzyme, converting acetaldehyde to ethanol in a NADH^+^H^+^-dependent reaction [13]. In *M. circinelloides*, the mRNA of *ADH1* gene is expressed in spores, hyphae, and yeast cells, and produces a cytoplasmic enzyme that appears to be the major Adh1 enzyme in this fungus [14]. The level of expression of the *ADH1* gene during aerobic mycelial growth is correlated with the concentration of glucose in the culture medium [13].

Oleaginous microbes have been found to accumulate extra lipids when grown under high glucose and low nitrogen conditions [6]. At high glucose concentrations, however, some other non-lipid biosynthetic pathways also became more active which resulted in the diversion of glucose to other metabolites. Keeping a focus on developing *M. circinelloides,* the strain was engineered with the intention to accumulate more lipids. Our previous finding indicates *M. circinelloides* strain WJ11 as a dominant producer of lipid; therefore, optimizing the production of lipid by diverting its metabolic flux of other pathway to towards the lipid biosynthesis is essential. Redirecting carbon metabolism from ethanol generation to fatty acid biosynthesis was one of the methods used in this investigation. To achieve this aim, *M. circinelloides* WJ11 open reading frame of alcohol dehydrogenase enzyme was deleted. The effect of the knockout of *adh1∆* gene on fatty acid profile and accumulation was investigated and discussed. Our results showed that ΔMcADH1 (mutant strain) had no prominent effect on growth under normal oxygen conditions, while it had impaired lipid and fatty acid production. The addition of exogenous ethanol restored the lipid production in the mutant strain of *M. circinelloides*. Our study suggests that ethanol produced under aerobic conditions by McADH1 (wild type) is important for the normal growth and lipid production in WJ11.

## 2. Materials and Methods

### 2.1. Strains and Cultivation Conditions

The uracil auxotrophic strain MU65 of *M. circinelloides* WJ11 (CCTCC No. M 2014424) was used as the host strain for the adh1 gene knockout study. The strain was maintained in the YPG media which contains 0.3% yeast extract, 1% peptone, and 2% glucose supplemented with uridine (200 μg/mL) [15]. The transformed colonies of MU65 were selected using MMC media (20 g/L glucose, 10 g/L casaminoacid, and 0.5 g/L yeast nitrogen base without amino acids) supplemented with 1 mg/L niacinamide and 1 mg/L thiamine chloride [16]. The pH of YPG and MMC media was adjusted to 3 and 4.5 for colonial and mycelial growth, respectively. For cloning and plasmid storage, the *E. coli* DH5α was used and cultivated in Luria–Bertani media supplemented with ampicillin with shaking 220 rpm at 37 °C [17].

### 2.2. Construction of adh1 Knockout Plasmid

Plasmid, pUC18, which contains the *M. circinelloides pyrF* gene surrounded by 5′ and 3′ up- and down-stream ∼1 kb of adh1 sequences, was used for the construction of knockout plasmid for the disruption of the adh1 gene. The assembled knockout (KO) cassette that contains *pyrF* encodes an enzyme for uridine as a selectable marker and flanking sequences corresponding to regions surrounding the adh1 gene allow its chromosomal integration by homologous recombination. The DNA sequences of Up, Down, and *pyrF* genes were amplified by PCR with corresponding primers F/R (Supplementary Table S1) from the genome of wild-type *M. circinelloides* WJ11. Up Stream (1000 bp), *pyrF* (1962 bp), Down Stream (1000 bp) fragments were ligated into 3962 bp single fragments by PCR overlap extension method and connected to the multiple cloning site 5′-SphI-SmalI-3′ of the vector pUC18 to form a recombinant plasmid UP STREAM-pyrF-DOWN STREAM-pUC18. The obtained plasmid was cloned in DH5α competent cells. Knockout (KO) cassette was released from the recombinant plasmid by double digestion with restriction enzymes (SphI and SmaI) and the released fragment was introduced into MU65 protoplasts for homologous recombination by electroporation-mediated transformation [18]. After subculturing method in the selective medium, transformed strains were isolated, confirmed, and validated by PCR.

### 2.3. Fermentation and Lipid Analysis of adh1 KO Strain

The K&R medium (glucose 80 g/L, diammonium tartrate 3.3 g/L, yeast extract 1.5 g/L, MgSO_4_∙7H_2_O 1.5 g/L, FeC1_3_∙6H_2_O 8 mg/L, ZnSO_4_∙7H_2_O 1 mg/L, CuSO_4_∙5H_2_O 0.1 mg/L, Co(NO_3_)_2_∙6H_2_O 0.1 mg/L and MnSO_4_∙5H_2_O 0.1 mg/L, KH_2_PO_4_ 7.0 g/L, Na_2_HPO_4_ 2.0 g/L, and CaCl_2_∙2H_2_O 0.1 g/L [19] was used as fermentation study. The spores of wild type and knockout strains (1 × 10^7^ spores/mL) were inoculated in 150 mL K&R medium held in 1 L baffled flasks with shaking at 130 rpm for 24 h (seed media) and then inoculated at 10% (*v*/*v*) into 1 L modified K&R medium (fermentation media) for 4 days. The fermentation was carried out in a 1.5-L fermenter, stirred at 600 rpm, with 1.0 vvm aeration, and pH-controlled at 6.0. Cultures of knockout and wild-type strains were collected at 24, 48, 72, and 96 h for biochemical analysis of the fermentation process. Glucose concentration in the culture was also measured using a glucose oxidase Perid-test kit (Shanghai Rongsheng Biotech Co., Ltd., Shanghai, China) according to the manufacturer’s instructions. Biomass was harvested on a weighed filter paper by filtration through a Buchner funnel under reduced pressure and washed three times with distilled water, frozen overnight at −80 °C, and then freeze-dried. The weight of the biomass was determined gravimetrically and dried by lyophilization. The collected dry cell biomass was subjected to lipid extraction and fatty acid analysis by a method described previously [20].

### 2.4. Effects of Supplemented Ethanol on Cell Growth and Lipid Production

For liquid culture, the seed culture was started by inoculating 100 μL spores (1 × 10^7^ spores/mL) in K&R media, incubated at 28 °C at 150 rpm for 24 h. A 10% (*v*/*v*) seed culture was inoculated into a nitrogen-limited medium (glucose concentration of 80 g/L) in a 1.5 L fermenter supplemented with different concentrations of ethanol. Maintaining the other nutrients in the K&R medium, different concentrations of ethanol were added to investigate its effects on cell growth and lipid production of knockout strain.

### 2.5. Alcohol Determination

With minor modifications, the alcohol determination was carried out as previously described [21]. Fungal growth in liquid K&R media under aerobic conditions was as described above. The cultures were collected every 24 h during a 4-day growing period and the supernatant was collected by centrifuging the cultures. For alcohol determination, a supernatant of 25 μL was mixed thoroughly with 1.0 mL semicarbazide buffer (pH 8.7) and 25 μL NAD+ solutions (Solarbio, Beijing, China). Alcohol dehydrogenase solution (5 μL) sourced from *Saccharomyces cerevisiae* (Solarbio, Beijing, China) was added to the mixture and then incubated at 25 °C for 30 min. The absorbance of the reaction mixture was read at 340 nm. The standard curve was generated using 0–0.04% (*v*/*v*) ethanol solutions.

### 2.6. Gene Expression Analysis by Reverse Transcription-Quantitative PCR (RT-qPCR)

To extract total RNA, the fermentation samples were harvested after 48 h and immediately frozen in liquid nitrogen. Total RNA was isolated with Trizol after grinding the mycelium under liquid N2. RNA was reverse transcribed to cDNA using ReverTra Ace qPCR RT Kit. Real-time quantitative PCR (RT-qPCR) was carried out in a LightCycle 96 (Roche) using the SYBR Green Realtime PCR Master Mix according to the manufacturer’s instructions, which were based on the 2^−ΔΔCt^ method. The RT-qPCR reaction conditions were as follows: 95 °C incubation for 10 min, then 95 and 72 °C, 30 s for 45 cycles. The actin gene expression level was used to normalize the mRNA expression level, and the results were expressed as relative expression levels. The details of primers used in this study are given in the previous research of our group [22].

### 2.7. Statistical Analysis

Statistical analysis was carried out using (GraphPad Prism 8.0.2. San Diego, CA, USA) The mean values and the standard error of the mean were calculated from the data obtained from three independent experiments.

## 3. Results and Discussion

### 3.1. M. circinelloides WJ11 Knockout Mutant for adh1 Gene

**Figure 1:**
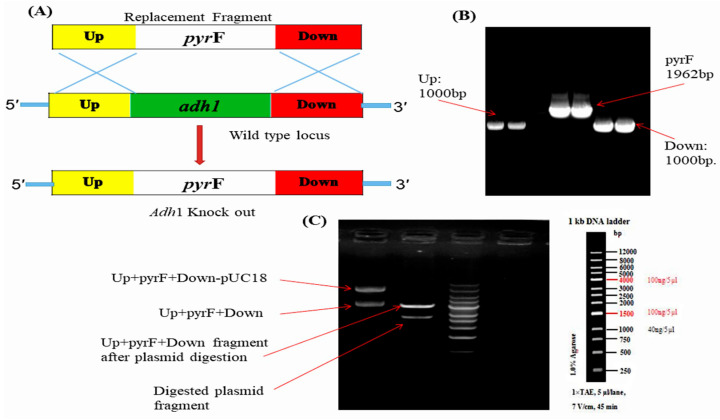
Construction of Knockout (KO) cassette for *adh1∆* gene deletion in *M. circinelloides* WJ11 (**A**) Homologous recombination event between replacement fragment and wild type locus; (**B**) PCR amplification of Up, *pyr*F, down gene from wild type; (**C**) Electrophoresis diagram of Up+pyrF+Down-pUC18 ligation and digestion verification.

Alcohol dehydrogenase I, encoded by *ADH1* gene, is a critical enzyme in fungal metabolism, catalyzing the conversion of acetaldehyde to ethanol. To investigate the role of alcohol dehydrogenase1 enzyme (Adh1) in the lipid production of *M. circinelloides*, the gene for Adh1 was deleted. There is a single gene—*ADH1*—in *M. circinelloides* WJ11 that codes for alcohol dehydrogenase enzyme, according to the available genomic data. The full length of cDNA of *M. circinelloides* WJ11 *ADH1* is 1048 bp (evm.model.scaffold00232.3) encodes a predicted protein of 348 amino acids residues containing typical ethanol dehydrogenase structural domains including Zn binding site and Adh activity domain. Previous studies have shown that, in the presence of high glucose under aerobic conditions, *M. circinelloides* produces significant amounts of ethanol, which shows that it is a Crabtree-positive microorganism [9]. Alcohol dehydrogenases play an important role in the metabolism of alcohols and participate in the last steps of fermentative metabolism or in the first steps of oxidative metabolism [12]. In *M. circinelloides*, it was observed that glucose positively regulates the production of the Adh1 enzyme and the kinetic characterization of purified Adh1 indicates that it acts as a fermentative enzyme and reduces acetaldehyde to ethanol [13]. Several characteristics of the oleaginous fungus *M. circinelloides*—including its well-established genetic tools, a clear genetic background, and ease of cultivation—make it an excellent candidate for the production of microbial lipids. *M. circinelloides* WJ11 is an ideal microorganism for studying lipid biosynthesis through metabolic engineering because of its complete understanding of its metabolism [7,23]. To determine if this gene was involved in fatty acid accumulation, a single deletion mutant was generated by gene replacement, designing a knockout vector to disrupt *ADH1* gene (Figure 1). The knockout vector for mutant contained the *pyr*F gene, flanked by ∼1 kb sequences of the adjacent regions of the corresponding gene (*ADH1* gene). Restriction fragments from the plasmid containing the whole construction were used to transform the MU65 strain of *M. circinelloides* WJ11, which is auxotrophic for uracil. Transformed strains were selected after repeated sub-culturing on the selective medium and were confirmed by PCR analyses (Figures S1 and S2).

### 3.2. Analysis of Cell Growth and Lipid Content in adh1 Knockout Strain

The knockout strains were grown in K&R medium for 96 h by both the shake flask method and in the fermenter. Previous study of the *ADH1* gene of *M. circinelloides* showed its high expression in cultures grown in a medium containing high glucose under aerobic conditions [13]. Therefore, for these conditions, K&R media containing high glucose 80 g/L was selected for the fermentation process. Lipid accumulation in oleaginous microbes is dependent on carbon metabolism and metabolic flux under particular conditions. *M. circinelloides* can grow effectively and accumulate a high content of lipid when the only carbon source is glucose [24]. The fermented broth at different intervals of fermentation time was tested to evaluate the impact of the deletion of the *adh1∆* gene on ethanol production under aerobic conditions. It was found that ethanol production increased in wild-type culture starting at 12 h of fermentation time and reached a maximum production level of approximately 5.8 g/L ethanol at 48 h of incubation. Our results are similar to the study conducted by Rangel et al. that reported 5 g/L ethanol at 48 h of aerobic fermentation in *M. circinelloides* [14], confirming that this fungus is a Crabtree-positive microorganism. However, in *adh*1 knockout strain after 48 h of fermentation time, the ethanol concentration was reduced by 85–90% compared to the wild type. The partial production of ethanol by mutant strain may be due to the end product of other metabolic pathways. McADH1 is primarily responsible for the conversion of acetaldehyde to ethanol in *M. circinelloides* under both aerobic and anaerobic conditions, as has been reported in other fungi [13,25]. This physiological function was similar to what had been observed in some fungi previously, such as *M. circinelloides* [13], *S. cerevisiae* [11,25], *Fusarium oxysporum* [26], *Candida maltose* [27], *Metarhizium* *acridum* [28], and *M. anisopliae* [21].

**Figure 2:**
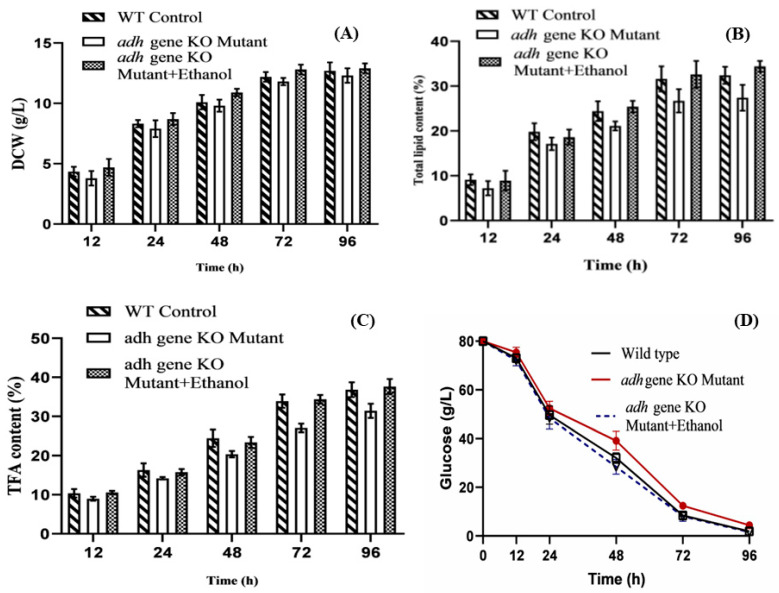
Comparison of dry cell biomass (**A**), total lipid production (**B**), total fatty acid content (**C**), and glucose utilization (**D**) of wild type, *adh*1 knockout mutant only, and *adh*1 knockout supplemented with 0.5% ethanol in batch cultures. Values were the mean of three independent fermentation experiments. Error bars represent the standard error of the mean.

During the entire fermentation process, the dry cell weights (DCW) and residual glucose in mutant cultures were measured to determine the effect of *adh*1 deletion on growth. The wild-type and knockout strains grew in a strikingly similar manner. When compared to wild-type levels, the DCW and glucose consumption rate of the knockout strain was slightly lower (Figure 2A,D). On the other hand, the accumulation of lipids in *M. circinelloides* WJ11 was significantly influenced by the deletion of the *adh1* gene. When compared to the wild-type control strain, the lipid and total fatty acid content of the knockout strain at 96 h of fermentation time was reduced by 15.4% and 14.66% respectively (Figure 2B,C). Remarkably, the total lipid and fatty acid content of the knockout strain was less, which confirmed that *adh1* deletion has a significant negative effect on the lipid accumulation in *M. circinelloides* WJ11.

Maintaining the other nutrients in the K&R medium, different concentrations of ethanol (0.1–1%) were added to investigate its effects on cell growth and lipid production in *adh*1 knockout strain. During this study, it was observed that supplementation of the culture medium with ethanol restored the lipid production of the *adh1∆* knockout strain. The cell growth, lipid, and fatty acid production of the mutant were restored at 0.5% ethanol in fermentation media (Figure 2A–C). There was a no significant difference in the production of biomass between wildtype and mutant; on the other hand, the difference in lipid and fatty acid content explains the ethanol content produced during the aerobic fermentation under high glucose concentration is important for physiological growth and lipid production. This study also revealed the importance of enzymes encoded by *ADH* gene in some microorganisms under aerobic conditions. *Adh*1 has been found to participate in the production of ethanol in *F. oxysporum*. Ethanol is used as a carbon source by conidia and mycelia, and *Adh* participates in this capacity and is required for the fungus to fully express its virulence [26]. When glucose is completely depleted and ethanol concentration reaches a maximum in *S. cerevisiae* and other Crabtree-positive yeasts, ethanol begins to be used as a carbon source, implying a close relationship between fermentative and respiratory metabolism [29].

### 3.3. Analysis of the Fatty Acid Composition of adh1 Knockout Strain

During the fermentation process the samples were collected at 96 h of fermentation time, and the resulting lipid from the biomass was subjected to GC analysis for fatty acid composition. The fatty acid profiles of the mutant strain showed that the knockout of *adh1∆* gene increased Gamma linoleic acid (GLA) while a decreased percentage was observed in palmitic acid, stearic acid, oleic acid, and linoleic acid fatty acids compared with the wild type strain (Table 1). This indicated that, despite the relatively lower total lipid content, a significant proportion of oleic acid had been transformed into useful polyunsaturated fatty acids throughout the lipid production process. The fatty acid profiles of the mutant strain supplemented with 0.5% ethanol showed that the GLA and the other fatty acid composition were restored similarly to the control strain. It is possible to use metabolic intermediates as medium supplements to boost the metabolic pathways that lead to the production of target products [30]. However, the role and mechanism of added metabolic intermediates on lipid synthesis in oleaginous filamentous fungi are still unclear at this point.

### 3.4. Fatty Acid Biosynthesis Gene Expression in ΔMcADH1

**Figure 3:**
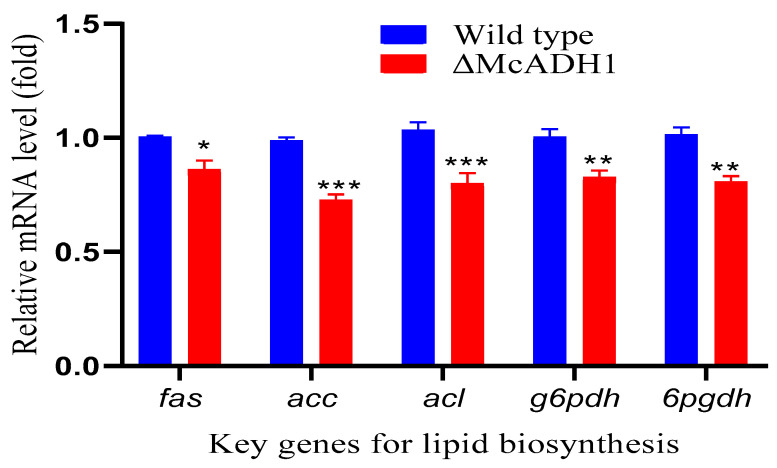
Lipid biosynthesis key genes expression in *adh1* knockout strain. RT-qPCR was used to determine relative mRNA levels of fatty acid synthase (*fas*); acetyl-CoA carboxylase (*acc*); ATP: citrate lyase (*acl*); glucose-6-phosphate dehydrogenase (*g6pdh*); 6-phosphogluconate dehydrogenase (*6pgdh*) at 48 h of fermentation time. The values are the means of three biological replicates and asterisks indicate significant differences: * *p* < 0.05, ** *p* < 0.01, *** *p* < 0.001.

The total lipid content of the *adh*1 mutant was dramatically impacted when compared to the control. As a result, we investigated the impact of *adh*1 deletion on the transcriptional level of key genes for fatty acid biosynthesis at 48 h, when ethanol production was high in the control group. To better understand fatty acid biosynthesis, we selected and studied the key genes that play a pivotal role in the various reactions of lipid accumulation in oleaginous microbes. Citrate lyase (acl), acetyl-CoA carboxylase (accA), fatty acid synthase (fasB), glucose-6-phosphate dehydrogenase (g6pdhA), and 6-phosphogluconate dehydrogenase (6pgdhA) were studied because they are key enzymes in the fatty acid synthesis pathway and they catalyze rate-limiting events for de novo fatty acid biosynthesis [22,31,32,33,34,35]. The transcriptional levels of fasB, accA, acl, g6pdhA, and 6pgdhA gene were slightly downregulated by the margin of 18%, 25%, 19%, 14%, and 17% respectively in the mutant as compared to wildtype (Figure 3). Given the considerable drop in lipid quantity in the mutants, we can predict that the deletion of the alcohol dehydrogenase could reduce all of the precursors required for the lipid production pathway. However, additional research is required to test this idea and fully comprehend the ethanol process in lipid accumulation in the oleaginous fungus *M. circinelloides*.

*M. circinelloides* produces γ-linolenic acid (GLA, C18:3 n-6; cis 6, 9, 12-octadecatrienoic acid), a nutritionally significant polyunsaturated fatty acid (PUFA) with favorable benefits on human and animal health. Researchers are attempting to uncover new sources of omega-3 fatty acids by developing modified lipid-producing microorganisms in order to meet the growing demand for these acids. There are many metabolic pathways involved, hence it is hard to regulate just one or two genes to promote maximum lipid accumulation. To obtain insights into the molecular mechanism of lipid accumulation, a rigorous examination of lipid metabolism is essential. By investing in the lipid accumulation mechanism, researchers can not only boost total lipid yields by promising *M. circinelloides* WJ11, but also expand the basic knowledge of lipid accumulation in oleaginous microbes.

## 4. Conclusions

In this study, we tried to investigate the *ADH1* gene of *M. circinelloides* which produces ethanol under aerobic conditions. Assuming that inhibition of ethanol production may be linked to high lipid production, we have conducted a study to knock out the gene responsible for ethanol production in *M. circinelloides* WJ11 by using the homologous recombination method. From the fermentation analysis of the knockout strain, it was found that the mutant strain produces low lipid and fatty acid content as compared to the wild-type control. We also observed the restoration of lipid and fatty acid synthesis in the mutant strain when the fermentation media was supplemented with ethanol, suggesting the importance of ethanol in the overall growth of *M. circinelloides*.

## Figures and Tables

**Table 1:** Fatty acid profile of Wild type (WT), Knockout strain (ΔMcADH1), and ΔMcADH1 + 0.5% ethanol in fed-batch culture.

Fatty Acid	Fatty Acid % WT	Fatty Acid % ΔMcADH1	Fatty Acid % ΔMcADH1 + 0.5% Ethanol
Palmitic acid	16.64 ± 0.3	14.88 ± 0.8	16.75 ± 0.1
Stearic acid	6.59 ± 0.1	4.96 ± 0.2	6.42 ± 0.4
Oleic acid	35.25 ± 1.3	31.37 ± 1.5	36.18 ± 1.1
Linoleic acid	17.15 ± 0.7	16.71 ± 0.3	18.59 ± 1.0
Gamma linoleic acid	12.83 ± 0.6	14.17 ± 0.2	13.06 ± 0.7

## Supplementary Materials

The following supporting information can be downloaded at: https://www.mdpi.com/article/10.3390/jof8090917/s1, Table S1: Cloning primers, name, and sequence for the deletion of *adh1* in *M. circinelloides* WJ11. Figure S1: Adh 1 mutants that were picked after transformation on selective media. Figure S2: Mutants were selected by integration of DNA fragments (carries marker *pyrF* gene to replace *adh*1 gene) onto the genome of uracil auxotrophic *M. circinelloides* was verified through PCR. Primer pairs forward primer of Up DNA (PY199F) and Reverse primer of mid of pyrF (Md PYR) generating 1.86 kb band size that confirmed *pyrF* gene integration into genome.
