# Supplementary material for: Investigating the Effect of Alcohol Dehydrogenase Gene Knockout on Lipid Accumulation in Mucor circinelloides WJ11
Source: J Fungi (Basel). 2022 Aug 29;8(9):917. doi: 10.3390/jof8090917 (PMC9503276; doi:10.3390/jof8090917)
Supplement: Supplementary file 1 [file jof-08-00917-s001.zip › jof-1868712-supplementary.pdf]

Table S1: Cloning primers, name and sequence for the deletion of adh1 in *Mucor circinelloides* WJ11.

Gene name	Primer name	sequence（5′ to 3′）
Up	PY199FT（primer F）	CCAGTGCCAAGCTTGCATGCCTGCAGCTCGAGTGACACCATATTCTTGACGTCTGAATG
	PY199R1（primer R）	GTATATGAATATGAATAAGCCCAAGATTACCATCTGCTTTAACTG
pyrF	PY199F1（primer F）	GGTAATCTTGGGCTTATTCATATTCATATACTAGTCCCTTTG
	PY199R2（primer R）	GTATATTTTATAAATTTCTGCTAAATCCAGATTTCACATGG
Down	PY199F3（primer F）	ATTTAGCAGAAATTTATAAAATATACATAGCACCCCCTTTTTGTAGC
	PY199RT（primer R）	GATTACGAATTCGAGCTCGGTACCCGGGCTTAGCTGGCCAGCAGCTATTTC

List of primer names and sequences for RT-PCR

RT-PCR for acl

GGAAAAGAAATCAAGATTGAAAGAACGACTG (F)

AGCCTTGGCATCAACATCACC (R)

RT-PCR for accA

GATATGCATTCCCGTGTTTCCG (F)

ACCGAGTAATTCATCAGCTGGG (R)

RT-PCR for fasB

GGTCTTCGTTTCATTGACCCCACTG (F)

GACATCAGCCTTGTCACCTTGTTGG (R)

RT-PCR for g6pdhA

ATTGTTTCACAGAGGAAGAGACTTTCC

TGTTATCAATGTTGGAACGGTTCCAG

RT-PCR for 6pgdhA

GATGGTTCACAACGGTATTGAATACGGA

CGATCAAGAAAGAATCCAATTCACCCTTG

RT-PCR for actin

GATGAAGCCCAATCCAAGAGAGGT

TCTTCTCCGGTTGGACTTGGG

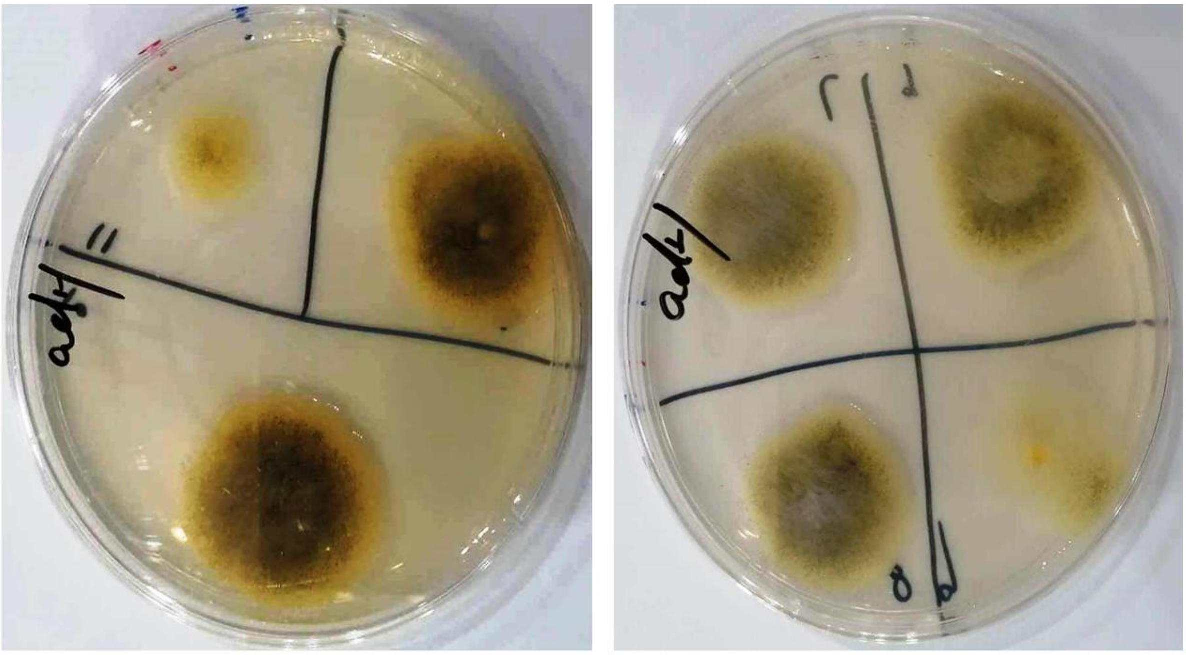

Figure S1: Adh 1 mutants that were picked after transformation on selective media.

PY199FT（primer F Forward primer of Up DNA as shown Supplementary table 1)

5′CCAGTGCCAAGCTTGCATGCCTGCAGCTCGAGTGACACCATATTCTTGACGTCTGAATG 5′

Md PYR (primer R designed from the mid of pyrF gene)

5′ GTGAGAGGCAGAGAACTCTTTGTAGT 3′

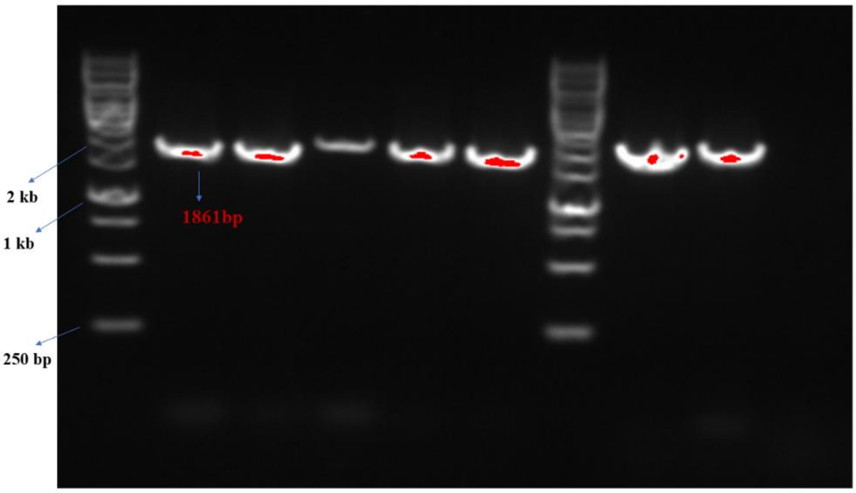

Figure S2: Mutants were selected by integration of DNA fragments (carries marker *pyrF* gene to replace *adh*1 gene) onto the genome of uracil auxotrophic *M. circinelloides* was verified through PCR. Primer pairs forward primer of Up DNA (PY199F) and Reverse primer of mid of pyrF (Md PYR) generating 1.86 kb band size that confirmed *pyrF* gene integration into genome.
